# 2-year survival and cost analysis of occlusoproximal ART restorations using encapsulated glass ionomer cement in primary molars: a randomized controlled trial

**DOI:** 10.1186/s12903-024-04357-9

**Published:** 2024-06-01

**Authors:** Jonathan Rafael Garbim, Cintia Saori Saihara, Isabel Cristina Olegário, Daniela Hesse, Mariana Pinheiro Araujo, Clarissa Calil Bonifácio, Mariana Minatel Braga, Daniela Prócida Raggio

**Affiliations:** 1 Department of Orthodontics and Pediatric Dentistry, School of Dentistry, University of São Paulo, Av. Lineu Prestes, 2227 São Paulo, Brazil; 2 Department of Public & Child Dental Health, Dublin Dental University Hospital, Trinity College Dublin, Dublin, Ireland; 3 Academic Centre for Dentistry Amsterdam (ACTA), Amsterdam, The Netherlands; 4 FDI (World Dental Federation), Geneva, Switzerland

**Keywords:** Pediatric dentistry, Dental atraumatic restorative treatment, Primary teeth, Clinical efficacy, Cost analysis, Glass ionomer cement

## Abstract

**Background:**

The survival of ART restorations can be influenced by the choice of the restorative material. The aim of this randomized non-inferiority controlled trial was to compare the 2-year survival rate and cost analysis of two encapsulated glass ionomer cements (GIC) as occlusoproximal restorative materials in primary molars.

**Methods:**

Children from public schools in Tietê (Brazil), aged 4–8 years with occlusoproximal dentine carious lesions in primary molars were selected and randomly assigned to receive either Equia Forte (EF) or Riva Self Cure (RSC) as restorative materials. Treatment was carried out by two trained final-year dental students in schools following ART premises. Restorations were assessed by a trained and calibrated examiner after 2, 6, 12, 18, and 24 months. The primary outcome was restoration survival after 2 years, analyzed using Kaplan-Meier survival and Cox regression analysis (α = 5%). Professional and materials costs for each group were collected in Brazilian Reais (R$) and converted into US dollars (US$) and analyzed using Monte-Carlo simulation.

**Results:**

A total of 152 children (76 per group) were included in the study, and 121 (79%) were evaluated after 2 years. The overall 2-year restoration survival rate was 39% (EF = 45%; RSC = 32%) with no difference between the groups. The baseline and 2-year total cost of restorations using RSC was lower when compared to EF (incremental cost: US$ 6.18).

**Conclusion:**

After two years of follow-up, Riva Self Cure shows comparable restoration survival rates to Equia Forte, being more cost-effective in the Brazilian perspective.

**Trial Registration:**

This randomized clinical trial was registered on ClinicalTrials.Gov - NCT02730000.

## Introduction

Atraumatic Restorative Treatment (ART) is a Minimal Intervention approach for managing dental caries [1]. It is a patient-friendly treatment that requires no electricity, running water, or aerosol-generating procedures, and can be provided outside of dental offices with similar effectiveness as in clinical settings [2, 3].

Glass ionomer cement (GIC) has become the most used material for ART due to its chemical, biological, and mechanical properties [4]. However, dosing and hand mixing may increase the risk of error during material preparation, and operator skill is a significant factor in restoration survival rate [2]. To reduce this risk, encapsulated dental cement has been introduced as an option for ART and has gained popularity among dentists [5]. Encapsulated GICs are pre-proportioned in a powder/liquid ratio defined by the manufacturer and mechanically mixed, eliminating the operator’s influence on the functional properties of the material, which is the primary advantage of this type of glass ionomer cement [5].

Randomized clinical studies have shown similar survival rates between encapsulated and hand-mixed GICs [6, 7], and laboratory studies have demonstrated that encapsulated GICs produce specimens with lower porosity and higher mechanical strength than hand-mixed specimens [8–11]. Despite the variety of encapsulated glass ionomer cements available, more evidence is needed to determine the best-suited material for occlusoproximal ART restorations, and cost-effectiveness data is currently lacking.

The cost-effectiveness of ART depends on the initial treatment cost, as well as the costs incurred during regular follow-up and the expenses associated with retreatment if the restoration fails [12]. In this study, we compared the cost-effectiveness of two types of encapsulated glass ionomer cements: Equia Forte^®^ (EF-GC Corp) and Riva Self Cure^®^ (RSC-SDI). Even though there are several similarities between the two materials (both are described by their manufacturer as a bulk-fill, fluoride releasing, glass hybrid restorative systems), EF has a higher market price compared to RSC.

The aim of this randomized non-inferiority clinical trial was to compare the 2-year survival rate and cost of two encapsulated GICs as occlusoproximal restorative materials in primary molars. The null hypothesis is that the RSC is non-inferior to EF in terms of treatment survival after 2 years.

## Materials and methods

This manuscript was written according to the recommended Consolidated Standards of Reporting Trials guideline (CONSORT) [13].

### Study Design and ethical consideration

This is a double-blind (participant and outcome assessor), randomized, non-inferiority, two-arm (1:1 allocation) clinical trial. The study was registered on ClinicalTrials.gov (NCT02730000–06/04/2016) and ethically approved by the Research Ethics Committee of the University of São Paulo School of Dentistry (CAAE number 54139615.9.0000.0075). Participants could only be included in the study after their parents/guardians gave written consent for their children to participate. Eligible children were asked to accept or decline participation using an assent form as their willingness to participate in the study.

### Deviations from the protocol

Our original intention was to stratify the sample based on caries experience; however, this stratification was not executed as planned.

### Sample size calculation

The sample size was based on the primary outcome - survival of encapsulated glass ionomer cement restoration in occlusoproximal cavities in primary molars. The sample size estimation was performed on the website https://www.sealedenvelope.com. A non-inferiority limit of 20% and a survival rate reported by Ersin et al., 2006 [14] for occlusoproximal ART restoration of 76% after 24 months was assumed for estimation. Considering a significance level of 5%, a power of 80%, and considering 20% for potential loss, we achieved a minimum sample size of 136 children. Only one tooth was included per child.

### Randomisation

The allocation sequence was generated electronically through a website (http://www.randomization.com/) with permuted block sizes (4, 6, and 8). The information was sealed in opaque envelopes and numbered sequentially.

Randomization was at the participant level, with children allocated to Equia Forte - Gc Corp (EF – Positive Control Group) or Riva Self Cure (RSC - Experimental Group). An independent dentist generated the allocation sequence, and eligible children were randomly allocated to the treatment groups. The envelope containing the proposed treatment was sequentially selected by the dentist and opened only when the child was ready to undergo treatment by one of the operators.

### Blinding

The blinding of the operators was not possible since both GIC capsules had different packaging and capsule colors. The outcome assessor was blinded to the study groups as restorations had similar clinical presentation.

### Eligibility criteria

Children aged 4 to 8 years attending public schools in the city of Tietê, Brazil, were screened and invited to participate in this study if they presented:

- at least one occlusoproximal caries lesion in dentin on a primary molar without signs or symptoms of pulpal involvement;
- generally cooperative behavior that could be managed by the operators in the school environment;
- no existing medical conditions.

Children who were considered eligible for the study were included only after the parents/guardians sent the signed informed consent form agreeing to their child’s participation in the study, and the child’s consent. We included only one tooth per child, and all treatments were performed in public school classrooms. If the child had more than one decayed tooth, a simple draw was made to select which tooth would be included. If the child needed additional treatment, they were referred to the nearest public oral health center.

### Interventions

The children were evaluated and treated during school hours in empty classrooms (no dental conventional facilities). Operators were two trained 5th year undergraduate dental students. A theoretical lecture on ART [15] including instructions of the treatment protocol to be used in the present study was given to the students. The training of the operators consisted in laboratorial practice with restorative materials using frasaco teeth followed by clinical exposure. The students had the opportunity to practice how to place ART restorations in children that were not participating in the present study prior to the start of the study.

All restorations were performed with relative isolation of the operative field. Selective caries removal to firm dentine was performed with hand instruments of appropriate size. The cavity was then cleaned with wet cotton pellets. The restorative protocol was the same for both materials:

- Dentine conditioning: cavity conditioner (RIVA conditioner or EQUIA Conditioner) was applied in the cavity using a microbrush for 10 s.
- Cleaning of the cavity: the dentine conditioner was washed out using three wet cotton pellets and the cavity was dried using three dry cotton pellets.
- Matrix band placement: a stainless steel sectional matrix band was placed and kept in position with the aid of a wooden wedge.
- Mixing of the GIC material: the GIC capsule (RSC or EF) was activated by pressing it down on a hard surface and mixed for 10 s using a mixer  (Ultramat SDI).
- Application: the GIC capsule was clicked three times with capsule applier (SDI) and dispensed directly into the cavity to be restored. The material was pressed using finger press technique and the excess material was removed using a probe. The matrix band was removed after material initial setting (approximately 5 min). Flossing was used to remove any excess material and check contact point reestablishment.
- Surface protection: a thin layer of the material coating system was applied over the final restoration (Equia Coat or Riva Coat) and light cured for 20 s.

Child’s full name, date of birth, school details and date of treatment were collected to contact children during the follow-up period. Other independent variables were also collected, including child-related data (age, sex, caries experience) and teeth-related data such as cavity size in mm^3^ (occluso-cervical/bucco-lingual/mesial distal dimensions measured using WHO probe), restored surface (mesial/distal) and GIC material (EF/RSC).

The time spent on each restoration session was recorded by an assistant researcher from the time the participant lay on the school table until the restoration was completed to calculate the duration and cost of treatment.

All children were instructed not to consume solid foods for one hour after treatment.

### Evaluation of restorations (primary outcome)

An independent, trained, calibrated (Kappa with reference standard = 0.84 and intra-examiner = 0.97) blinded regarding groups examiner assessed the restorations using the Roeleveld el al. [16] criterion after 2, 6, 12, 18 and 24 months. Scores of 00 and 10 were considered success while scores of 11, 12, 13, 20, 21, 30 or 40 were considered failure. Scores of 50, 60, 70, or 90 were censored in the survival analysis. All recorded data were considered in individual clinical records for statistical analysis.

### Cost estimation (secondary outcome)

Costs for each group were estimated using a micro-cost approach, accounting for professional and material costs (payer perspective). All costs were measured in Brazilian Real (R$) and converted into US Dollars (US$).

To calculate the professional cost, the session time was timed by a researcher (other than the operator) including return visits. Thus, the time spent in each session was converted into hours and multiplied by the average income of the dentist per hour (US$ 12.97) and of a dental assistant (US$ 7.41), according to the Brazilian Federal Law 3991/61 [17]. For uncountable products, an estimate was made based on their production and divided by the average value of each package. For countable materials, the number of items in each package was divided by the total price of the product. Costs such as housing and municipal taxes were not considered. No discount rates were applied. Only one failure per restoration was considered for analysis. All data was tabulated in Excel.

### Statistical analysis

Analysis of the primary outcome was compared using a non-inferiority two-sample test for survival data using Cox Regression (non-inferiority hypothesis/alternative HR > 0.80; CI = 90%). Considering the proportion of treatment success at 2-year follow-up, an intention-to-treat analysis (using multiple imputations considering baseline variables) was conducted as a sensitivity analysis using the p-value non-inferiority test and confidence interval (CI = 95%). These analyses were performed using NCSS statistical software (NCSS 2021, USA).

As a secondary analysis, a Cox regression analysis was performed to investigate the association of other independent variables and restoration failure (two-tailed p values were reported). Treatment survival was assessed using Kaplan-Meier survival analysis and the Log-rank test (α = 5%). Baseline and total 2-year incremental cost between groups were compared using linear regression analysis considering the child level, and Bootstrap replications were set to 1000 using Stata 13.0 software.

For the cost-effectiveness analysis a Monte-Carlo simulation was performed according to the survival values of each material to calculate the variables ΔT (survival time) and ΔC (incremental cost), representing the difference in months between the survival time rate of restorations with EF and RSC, and the difference between treatment costs. The number of simulations was set to 10,000, the variables ΔT and ΔC were computed using XLSTAT 2018. Finally, the values of ΔT and ΔC were plotted on two cost-effectiveness planes (scatter plots).

## Results

**Fig. 1:**
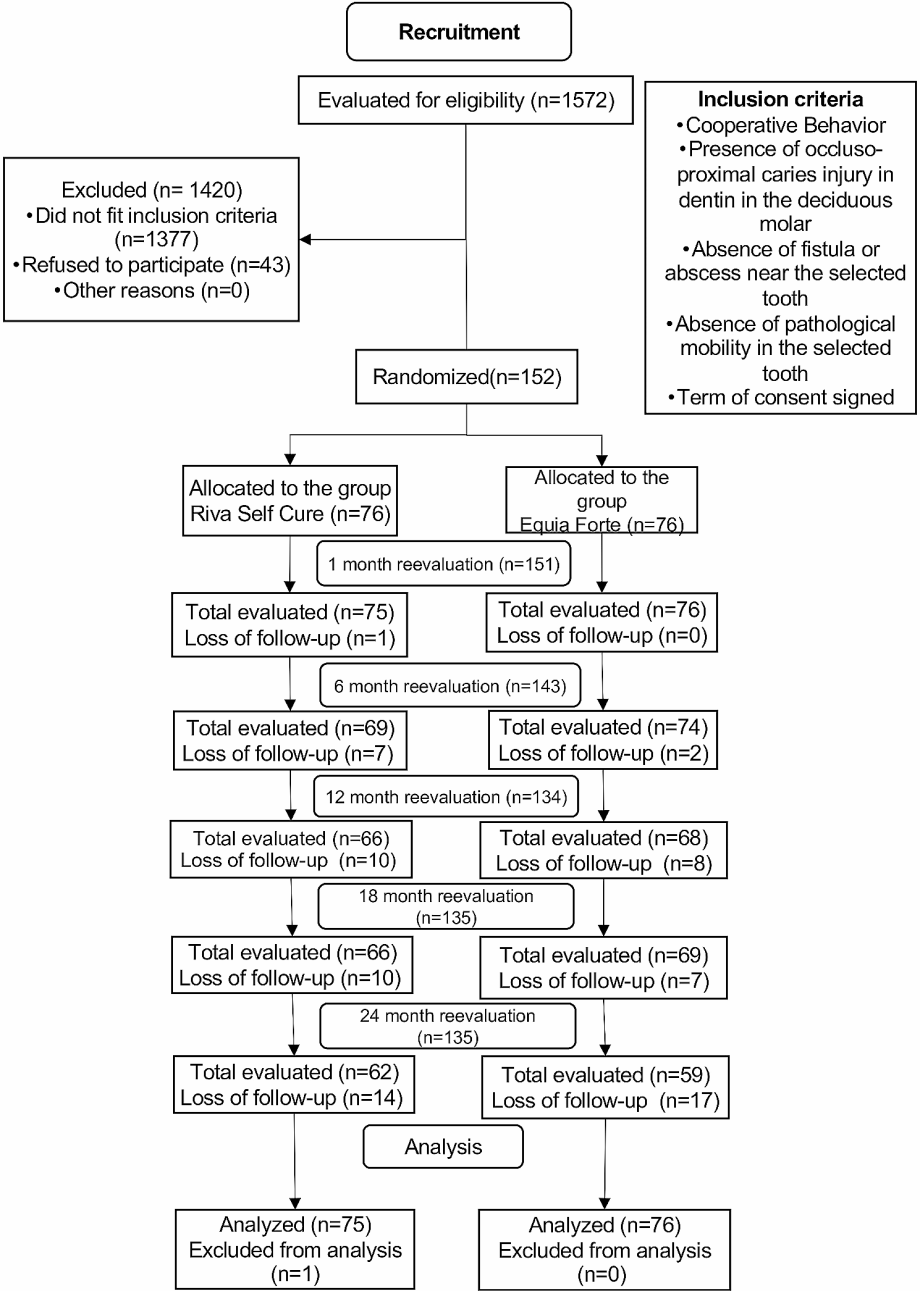
CONSORT flowchart for clinical trials. *Reasons for loss of research follow-up*: Transfer to another school in another state or city and/or absence on the day of reevaluation; *Inclusion in the analysis*: The research participant has come to at least one evaluation; *Exclusion of the analysis*: The research participant is not present on the day of the reevaluation

A total of 1,572 children between 4 and 8 years old were screened in 16 different public schools in the municipality of Tietê-SP in July 2018, and 152 were considered potentially eligible and invited to participate in the study. The children were randomly allocated (76 to the EF group, and 76 to the RSC group). Only one tooth was included per child. The CONSORT flow chart for clinical trials is shown in Fig. 1.

Among the 152 children included in this study, most of the participants were boys (56%). In total, 65 molars were maxillary (43%) and 86 mandibular (57%). The main reason why children were not included was because they did not meet our eligibility criteria (*n* = 1420), while 43 children refused to participate in the study. No children switched to the other group during the trial. More information about the basic characteristics of the participants is available in the additional file, and the descriptive analysis of the independent variables equivalent to the restorative material (EF and RSC) are presented in Tables 1 and 2, respectively.

**Table 1:** Description of the restorative materials

Groups	Restorative Material	Composition	Expiry date / Batch
**Experimental** Capsule	**SDI** **Riva Self Cure** ^**®**^	Fluoraluminosilicate glass (92 to 97%) Polyacrylic acid (3 a 8%)	2018-01 / B1510291F
**Control** Capsule	**GC CORP** **Equia Forte** ^**®**^	Fluoraluminosilicate glass (90 to 100%) Polyacrylic acid (5 a 10%)	2017-04 / 1,504,211

**Table 2:** Descriptive analysis of the independent variables by restorative material (Equia Forte and Riva Self Cure)

Variables	Equia Forte *n* (%)	Riva Self Cure *n* (%)	*p*-value Chi-square	Stayed in *n* (%)	Dropped-out *n* (%)
***Operator***					
1	38 (50.67)	37 (49.33)		59 (78.67)	16 (21.33)
2	38 (49.35)	39 (50.65)	0.871	62 (80.52)	15 (19.48)
***Caries Experience (DMFT/dmft)***					
≤ 3	16 (38.10)	26 (61.90)		29 (69.05)	13 (30.95)
> 3	60 (54.55)	50 (45.45)	0.070	92 (83.64)	18 (16.36)
***Jaw***					
Upper	34 (52.31)	31 (47.69)		51 (78.46)	14 (21.54)
Lower	42 (48.28)	45 (51.72)	0.623	70 (80.46)	17 (19.54)
***Age (years)***					
3–5	18 (45.00)	22 (55.00)		28 (70)	12 (30)
> 5	58 (51.79)	54 (48.21)	0.461	93 (83.04)	19 (16.96)
***Sex***					
Female	37 (55.22)	30 (44.78)		50 (74.63)	17 (25.37)
Male	39 (45.88)	46 (54.12)	0.253	71 (83.53)	14 (16.47)
***Volume***					
≤ 10mm^3^	14 (46.67)	16 (53.33)		23 (76.67)	7 (23.33)
> 10mm^3^	62 (50.82)	60 (49.18)	0.684	98 (80.33)	24 (19.67)
***Surface***					
OM	28 (45.16)	34 (54.84)		48 (77.42)	14 (22.58)
OD	48 (53.33)	42 (46.67)	0.322	73 (81.11)	17 (18.89)
**Total**	76 (50)	76 (50)		121 (79.61)	31 (20.39)

### Outcome evaluations

**Fig. 2:**
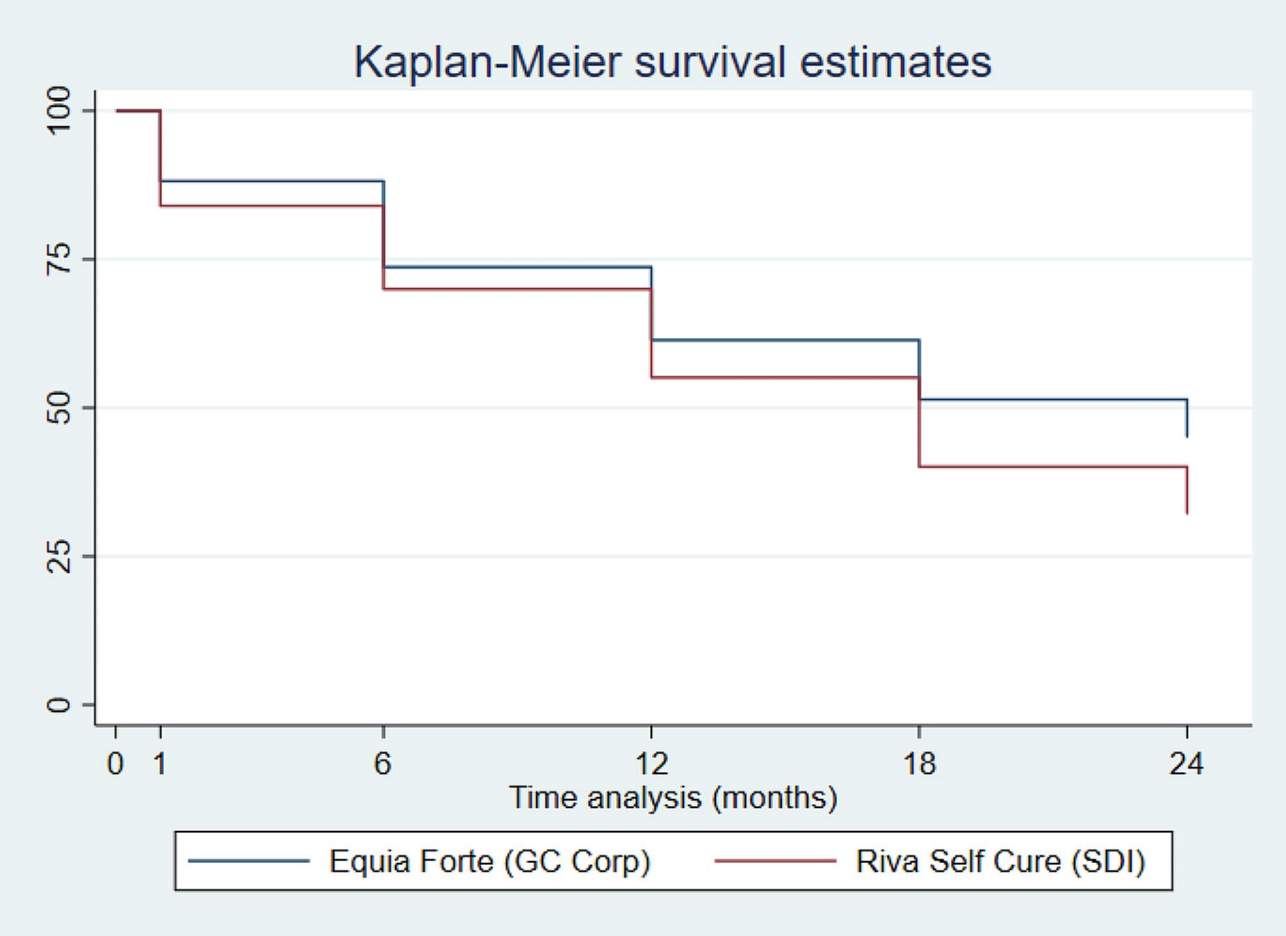
Kaplan-maier survival analysis

One hundred and twenty-one children (79%) had the study tooth evaluated after 24 months and 31 children (21%) were lost to follow-up. The survival after 24 months was EF 45% and RSC 32% (log-rank *p* = 0.020). The alternative hypothesis of non-inferiority was accepted by both the Cox regression analysis and the intention-to-treat analysis (EF = 33%; RSC = 30%; *p* = 0.002). The Kaplan-Maier survival plot, the primary outcome analysis using Cox non-inferiority regression and the ITT analysis can be found in Fig. 2; Table 3, respectively.

**Table 3:** Primary outcome analysis (restoration survival) using non-inferiority Cox Regression and Intention-to-treat analyses

Outcomes	EQUIA	RIVA	*p*-value
Primary outcome – Non-Inferiority Cox Regression analysis*			
% Survival	32%	45%	0.020*
HR (90% C.L. of HR)	1.25 (0.88–1.79)		
Primary outcome – Intention-to-treat analysis (2 years) **			
N success/N total	25/76	23/76	0.002*
% Success	32.9%	30.2%	
Absolute difference (95%CI)	0.026 (-0.12 to 0.173)		

HR = Hazard Ratio

Ha = non-inferiority at α = 5%

* 100(1–2α)% Confidence Interval and p-value for non-inferiority survival data (Wald test)

** p-values and 95% CI were derived by Miettinen and Nurminen’s method using non-inferiority test for two proportions

The reason for the failure of the evaluation of the restorations was mainly related to fracture of the restoration (score 30), followed by the absence of the patient on the day of reevaluation (score 90). According to the univariate cox regression analyses, there was no statistical relationship between the independent variables (operator, caries experience, arch, age, gender, restoration volume and restored surface) and the survival of the restorations (Table 4).

**Table 4:** Univariate cox regression analyses between restoration failures and associated factors

Variable	2-year Survival%	SE	HR Univariate † 95% CI ‡	*p*-value
***Restorative material***				
Equia Forte (ref)	44.98	0.06	1.31 (0.85–2.01)	0.215
Riva Self Cure	32.06	0.06		
***Operator***				
1 (ref)	37.81	0.06	0.98 (0.63–1.51)	0.929
2	39.83	0.06		
***Caries Experience (DMFT/dmft)***				
1–3	34.86	0.08	0.88 (0.55–1.42)	0.616
> 3	40.24	0.05		
***Jaw***				
Superior (ref)	41.50	0.07	1.08 (0.70–1.68)	0.707
Inferior	36.56	0.06		
***Age (years)***				
3–5	34.52	0.08	0.78 (0.48–1.26)	0.319
> 5	40.11	0.05		
***Sex***				
Female (ref)	45.99	0.07	1.12 (0.72–1.74)	0.597
Male	34.75	0.06		
***Volume***				
0–10 mm^3^ (ref)	43.27	0.11	1.29 (0.73–2.29)	0.388
> 10mm^3^	37.55	0.05		
***Surface***				
OM	39.13	0.07	1.09 (0.70–1.69)	0.688
OD	38.22	0.05		
**TOTAL**	38.73	0.04	-	

HR = Hazard ratio; CI = confidence interval; SE = standard error 95% CI

The cost-effectiveness evaluations of the restorations were performed at 2, 6, 12, 18 and 24 months by measuring the time spent on each procedure, including costs such as consumables and professionals. Statistical analysis for the association of costs and treatment of the variables collected was performed using the Bootstrap Regression test. An incremental value for failure (score 11 and 12) or replacement (score 20, 21 and 30) of the restorations was added, stipulated at 50% and 100% of the total cost, respectively.

Initially the cost of RSC (US$12.73) was lower compared to EF (US$17.25), after 24 months the cost of EF continued to show a significant difference compared to RSC (*p* < 0.05). It is possible to see the difference in the final cost of US$6.18 over time when comparing EF to RSC. Other differences such as amounts spent on treatment as well as reevaluations can be found in the Table 5.

**Table 5:** Evaluation of the cost between materials over time using Bootstrap Linear regression analysis (1,000 repeats)

	Prospected mean U$ Dollar (SD)	Coefficient (SD)	*p*-value	95% Confidence Interval
**Baseline Total Cost**				
Equia Forte (ref)	17.25 (4.14)			
Riva Self Cure	12.73 (3.49)	-4.51	< 0.001*	-5.70 to -3.32
**6-months Total Cost**				
Equia Forte (ref)	21.24 (10.63)			
Riva Self Cure	15.26 (6.13)	-6.08	< 0.001*	-8.81 to -3.34
**1-year Total Cost**				
Equia Forte (ref)	23.11 (11.32)			
Riva Self Cure	16.74 (7.27)	-6.37	< 0.001*	-9.38 to -3.36
**18-months Total Cost**				
Equia Forte (ref)	25.17 (11.06)			
Riva Self Cure	18.57 (7.73)	-6.60	< 0.001*	-9.59 to -3.61
**2 years Total Cost**				
Equia Forte (ref)	25.48 (11.72)			
Riva Self Cure	19.30 (8.17)	-6.17	< 0.001*	-9.45 to -2.90

SD = Standard Deviation; *p < 0.05 95% CI

**Fig. 3:**
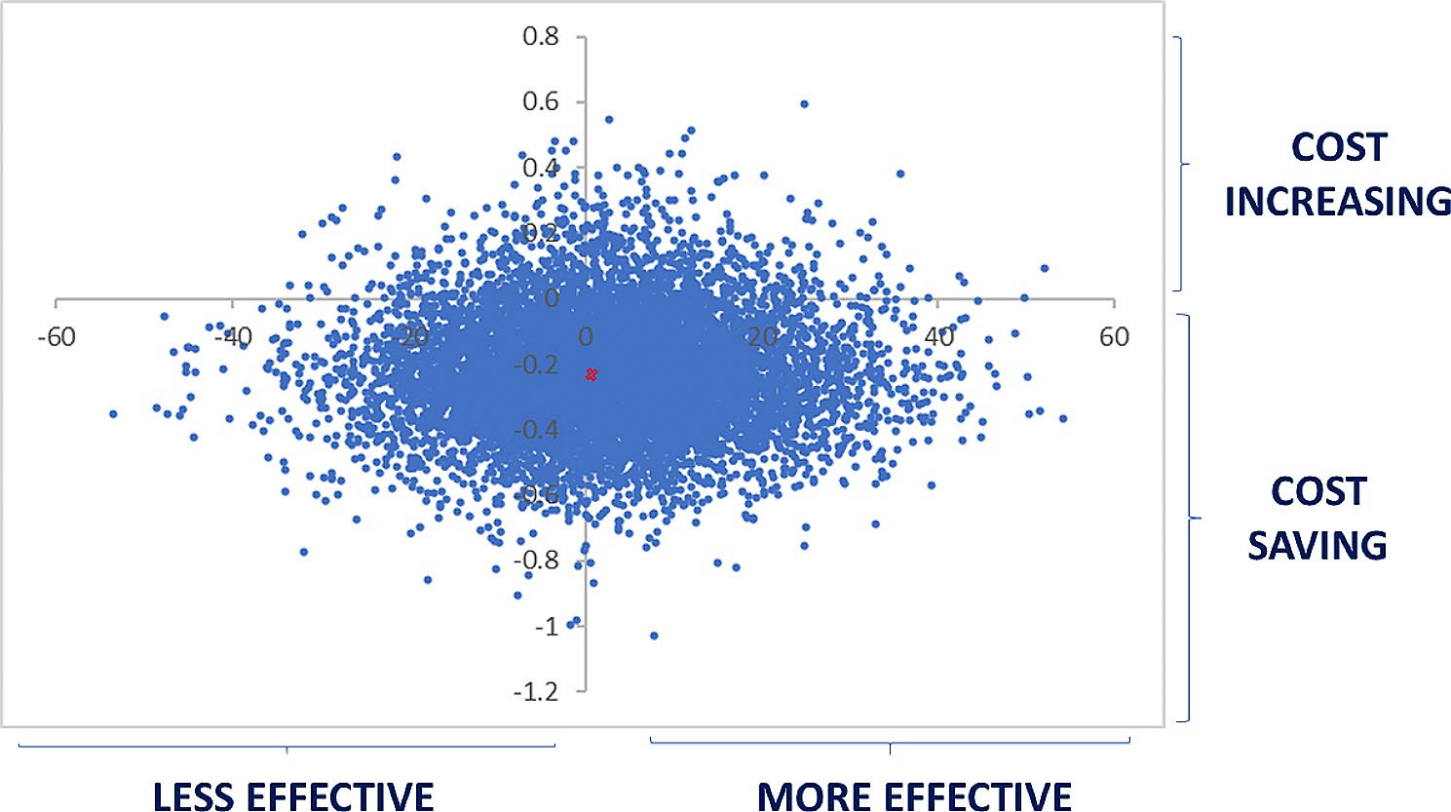
Riva self cure cost effectiveness plan related to Equia Forte reference material

All cases that required treatment due to restoration failure were referred to a health center, as explained previously by the Consent Form, these costs were stipulated and increased for the calculation of each reassessment. During the 24 month reassessment, the cumulative costs impacted by the material used were RSC US$19.30 and EF US$25.48 when compared to the initial cost. The cost-effectiveness plan confirmed the lower standard of effectiveness of EF when compared to RSC in occlusoproximal restorations in deciduous teeth and can be visualized in the scatter plot (Fig. 3).

## Discussion

The aim of this study was to investigate the cost-effectiveness of two brands of encapsulated GIC in ART restorations in occlusoproximal cavities of deciduous molars. As a result, we found that RSC was more cost-effective when compared to EF after 24 months.

A recent systematic review explored various factors influencing the survival of Atraumatic Restorative Treatment (ART) restorations [2]. Their findings highlighted the significant roles of operator experience and the number of restored surfaces in the long-term success of restorations. Conversely, another systematic review, published in the same year [3], reported no discernible differences in restoration survival between students and dentists. The operators in our study were final-year undergraduate dental students with limited experience in treating children. However, since the same operators, with consistent experience levels, performed all treatments in both restorative groups, their level of experience did not influence the primary study aim, which was to compare the survival of materials. To mitigate potential operator biases, the operators underwent training in ART restoration procedures based on the study protocol and received continuous supervision from a pediatric dentist (ICO/CSS). These supervising dentists were responsible for confirming eligibility criteria and diagnoses, ensuring adherence to the study protocol, and offering behavioral support during treatment. The use of encapsulated forms for both Glass Ionomer Cement (GIC) materials contributed to heightened standardization, reducing the likelihood of errors during powder/liquid mixing and GIC manipulation.

To evaluate if there were any variables that could influence the survival outcome of both materials, information such as caries experience, operator, arch, age, gender, restoration volume and restored surface were collected. The Cox regression analysis revealed no impact of any of the examined factors on the survival of the restorations. Similarly, the children who did not present at the 24-month reevaluation also did not influence the final analysis, since the Cox regression adjusted the data generating values from the parameters of this study.

A recent systematic review proved by a meta-analysis that there is no difference in the success rate of the ART technique when performed in the field and in the clinic. This reaffirms the results of this randomized clinical trial conducted in schools. In addition, to decrease a common problem with clinical trials, schools were notified of when evaluations would be performed, thus decreasing the chances of participants being lost to follow-up [3]. It is important to acknowledge that, similar to any randomized clinical study, our work operated within controlled conditions, potentially leading to an overestimation of the results. Additionally, in a pragmatic study, the longevity of the technique may be subject to the influence of uncontrolled factors, such as the child’s behavior.

A comparative study of occlusal and occlusoproximal restorations using the ART technique with high-viscosity glass ionomer cement found a 15% success rate for occlusoproximal restorations, unlike our study that found a 39% success rate after 24 months of follow-up [18]. This may be due to the different materials used, as the authors used manually hand mixing glass ionomers, and we used encapsulated ones.

The literature underscores elevated success rates associated with the use of encapsulated materials, akin to the material employed in our study. Miletić et al. (2020) [19] reported an impressive 93% success rate in their occlusal restorations utilizing encapsulated material (EF), while Freitas et al. (2018) [7] achieved a commendable 76% success rate in ART restorations in the posterior region using a different encapsulated material (RSC). These investigations showcase prolonged longevity for ART restorations compared to our study’s outcomes. This discrepancy may be attributed to tooth-related factors, considering both investigations were conducted in the permanent dentition. Existing research indicates that permanent teeth tend to exhibit greater longevity for ART compared to deciduous teeth [20]. Furthermore, Miletić et al.‘s study [19] specifically evaluated single-surface cavities, supported by scientific evidence indicating higher longevity for such restorations compared to occlusoproximal ones [20].

The study was conducted in a school setting, so general variable costs such as electricity, depreciation of equipment and instruments were not included, only direct costs such as professional costs and materials were evaluated, which may overestimate our cost since restorations were not repeated even when necessary. Also, since we estimated the final cost statistically, the result may not truly represent the actual final cost. Our conclusions can be transferred to other healthcare systems if it is done sparingly and considering the context of the healthcare system applied. We also do not account for the costs of experimentation and implementation. However, these are likely to be limited, since ART is easy to apply without specific equipment or a large amount of training. However, future studies should consider these costs. And these may be a limitation for this trial and the data analyzed.

The Monte-Carlo simulation, which performed a projection for a statistical sample of 10,000 simulations, allowed us to evaluate the cost-effectiveness of the treatment. In countries where the budget for human resources and material purchase are limited for both public and private health practices, selecting the material that offers the best balance between financial resource and effectiveness becomes crucial [21].

While the survival results have broader implications, our cost analysis was grounded in the Brazilian context, considering both professional and material costs. Despite the initial higher cost associated with EF compared to RSC, and the absence of significant differences in survival after a 24-month evaluation, the cost of EF remained consistently higher than that of RSC. This has notable implications for the Brazilian public service. The calculated incremental cost amounted to U$ 6.81 more per restoration, a considerable unit cost within the Brazilian context. Given the extensive volume of treatments administered in the country, implementing this finding could result in substantial savings in public funds. The cost-effectiveness ratio reinforces the conclusion that the Riva Self Cure restorative material demonstrates a dominant cost pattern, even though the survival rates are comparable for both groups.

## Conclusion

After 24 months of follow-up, the restoration survival from Riva Self Cure is non-inferior to Equia Forte. Additnionally, Riva Self Cure lexhibited a lower overall cost, establishing it the most cost-effective choice for occlusoproximal ART restorations in deciduous molars.

## Data Availability

The datasets generated and/or analyzed during the current study are available to the public upon request to the corresponding author.
